# Recombinant Antibodies in Veterinary Medicine: An Update

**DOI:** 10.3389/fvets.2018.00175

**Published:** 2018-07-27

**Authors:** Lorena Bustamante-Córdova, Edgar A. Melgoza-González, Jesús Hernández

**Affiliations:** Laboratorio de Inmunología, Centro de Investigación en Alimentación y Desarrollo, Hermosillo, Mexico

**Keywords:** recombinant antibodies, biotechnology, veterinary medicine, single-chain antibodies, nanobodies, chimeric antibodies

## Abstract

The production of recombinant antibodies has had a tremendous impact on several research fields, most prominently in biotechnology, immunology and medicine, enabling enormous advances in each. Thus far, a broad diversity of recombinant antibody (rAb) forms have been designed and expressed using different expression systems. Even though the majority of rAbs approved for clinical use are targeted to humans, advances in veterinary medicine seem promising. The aim of this mini-review is to present an update regarding the rAbs in veterinary medicine reported to date, as well as their potential use in diagnostics, prophylaxis and therapeutics. Full- and single-chain fragment variables are the most common forms of rAbs developed for the detection, prevention and control of parasitic, bacterial and viral diseases, as well as pain and cancer treatment. Nonetheless, advances in research seem to be skewed toward economically important animals, such as pigs, cows, poultry and dogs. Although significant results have been obtained from the rAbs reported here, most have not been developed enough to be approved. Further research and clinical trials should be encouraged to enable important findings to fulfill their intended potential to improve animal well-being.

## Introduction

Biotechnology has allowed for alternative methods of antibody (Ab) production, thereby reducing or eliminating the use of experimental animals (1). The selection of an expression system, such as bacteria, yeast, insect and mammalian cell lines, and transgenic plants (2, 3), depends primarily on the type of Ab desired. Frenzel et al. (4) published an excellent review on the expression of recombinant antibodies, discussing the pros and cons of the most-used systems (4).

Abs are glycoproteins that consist of two heavy (H) and two light (L) chains united by disulfide bonds. Light and heavy chains have a variable (V) and a constant (C) region; in turn, the constant heavy domain (C_H_) is divided into three domains (C_H1_-C_H3_) and in some cases, four domains. Variable regions contain “complementarity determining regions” (CDR) that determine the affinity and specificity of an Ab. The union of variable domain of light chain-constant domain of light chain (V_L_-C_L_) and variable domain of heavy chain-constant domain of heavy chain (V_H_-C_H1_) form the “antigen-binding fragment” (Fab). The rest of the Ab forms the fragment crystallizable region (Fc) and gives the Ab its effector function (5). The above characteristics represent the classical structure of an Ab.

**Figure 1:**
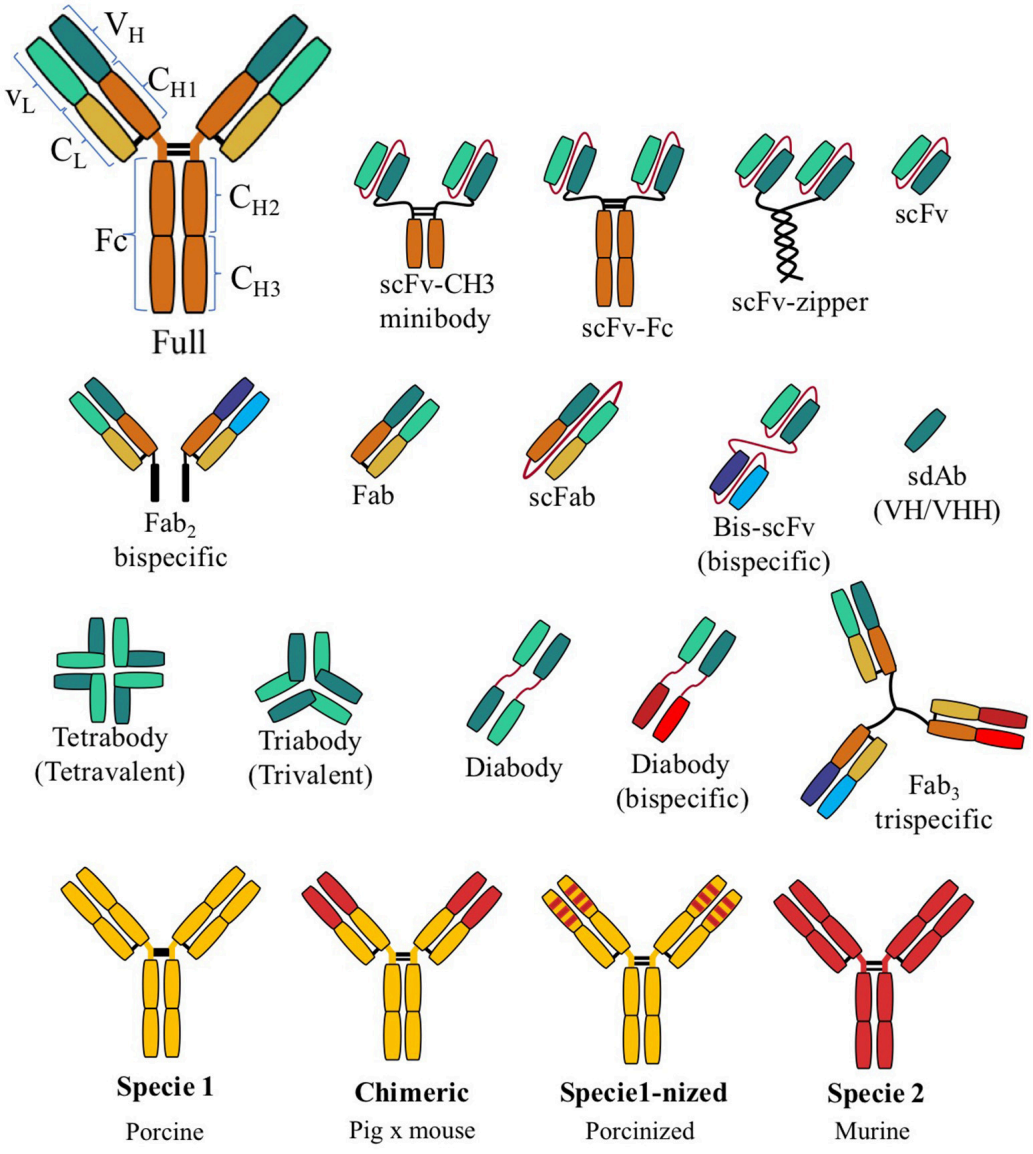
Graphic illustration of the diversity of recombinant antibody formats compared to a full, classic antibody. Abbreviations: variable domain of light chain (V_L_), variable domain of heavy chain (V_H_), constant light domain (C_L_), constant heavy domain (C_H_), single-chain fragment variable (scFv), antigen-binding fragment (Fab), fragment crystallizable region (Fc).

Based on the classical structure of an Ab, other forms of rAb have been developed (Figure 1). The most popular in veterinary medicine seems to be the single-chain fragment variable (scFv). The scFv comprises the V_L_-V_H_ joined by a short peptide linker (6). Another highly reported rAb is the single-domain antibody (sdAb) or nanobodies, which present the heavy variable region (VHH) only (7). Other rAbs, such as triabodies and tetrabodies (2), are not particularly reported in veterinary medicine (Figure 1). Hybrid rAbs, chimeric or “–nized,” are common in veterinary medicine. A chimeric Ab typically consists of the V regions of one Ab and the C region of another. The “–nized” Ab comprises the original Ab and CDRs from other species, such as humanized or porcinized. The main advantage of a hybrid rAb is reduced immunogenicity while maintaining the specificity of CDRs (8).

Orthoclone (Muromonab-CD3) was the first monoclonal Ab (mAb) approved in humans (9). Since then, Abs have become predominant products in the human pharmaceutical market (10). Since 2015, 23% of the drugs approved by the Food and Drug Administration in USA have been Abs, including humanized and chimeric, with just a few used in animals. This review presents an update of the rAbs reported in the field of veterinary medicine according to their use in diagnosis, prophylaxis and therapeutics in different species. However, there is an emphasis on rAbs in pigs and cows because they represent more prolific fields. Table 1 summarizes all rAbs described in this mini-review, showing the expression system, form, target species and main results. A discussion of the results describing only rAb production was omitted in the following sections.

**Table 1:** Summary of mentioned recombinant antibodies with potential use in veterinary medicine.

**Application**	**Ab format**	**Expression system**	**Species**	**Main result**	**Reference**
Diagnosis	sdAb	*E. coli*	Pig	High specificity and affinity for the capsid protein of PVC type II.	(11)
	sdAb	*E. coli*	Pig	High sensitivity and specificity recognizing the capsid protein of PCV type II by ELISA.	(12)
	Porcinized full Ab	HEK293	Pig	Recognizes the E2 protein of CSFV.	(13)
	scFv	*E. coli*	Pig	Recognition of *B. hyodysenteriae* for the diagnosis of swine dysentery.	(14)
	sdAb	*E. coli*	Pig	Specific recognition of *T. solium* antigens for the control of cysticercosis.	(15)
	scFv	*E. coli*	Cow	Binds to the 3B region of 3ABC non-structural protein of FMDV.	(16, 17)
	scFv	Plants	Cow	scFv directed at the VP1 protein of FMDV.	(18)
	scFv	*E. coli*	Cow	Highly specific to the capsid protein of BIV.	(19)
	scFv	HEK293	Cow	Detection of HSP65 from *M. tuberculosis*.	(20)
	sdAb (VHH)	*E. coli*	Cow	Recognizes *B. melitensis* strain Riv1 by ELISA.	(21)
	Full Ab	CHO	Cow	Higher sensitivity to recognize the pathogenic isoform of prion protein (PrP^sc^) than its scFv form.	(22)
	scFv	*E. coli*	Chicken	Specific to the NP of AIV.	(23)
	scFv	*E. coli*	Birds	Targeted to P phosphoprotein of NDV involved in transcription and replication.	(24)
	scFv	*E. coli*	Chicken	Differential diagnosis of classical and very virulent strains of IBDV.	(25, 26)
	scFv	*E. coli*	Chicken	Recognizes oocyst and macrogamont stages of *E. tenella* for the diagnosis of avian coccidiosis.	(27)
	Fc-sdAb (Fc-VHH)	*E. coli*	Dog	Recognizes Canine EGFR to label breast cancer cells.	(28)
Prophylaxis	scFv-Fc	HEK293	Pig	Antigen targeting of important structural peptides for PEDV and PRRSV to dendritic cell receptors.	(29, 30)
	Full Ab	HEK293	Pig	Antigen targeting of the glycoprotein 4 of PRRSV to CD169 receptor.	(31)
	sdAb (VHH)	*E. coli*	Pig	Binds to non-structural protein 9 of PRRSV.	(32)
	Chimeric M x P Full Ab	S*f*9 cells	Pig	Recognizes PRRSV glycoprotein 5 by WB and ELISA, neutralizing activity *in vitro*.	(33)
	scFv-Fc	HEK293	Pig	Antigen targeting to Langerin receptor. Enhances humoral and T CD4 responses against PEDV.	(34)
	scFv-Fc	HEK293	Pig	Reduction of RNA from PEDV in feces.	(35)
	scFv	*E. coli*	Pig	Neutralizing activity of PEDV *in vitro*.	(36)
	scFv	*E. coli*	Pig	Recognizes the recombinant C subunit of pAPN by ELISA.	(37)
	Fused sdAb with pIg (VHH2)	Yeast	Pig	Neutralizing activity *in vitro* of FMDV and reduced viremia *in vivo*.	(38)
	VHH3s	Yeast	Pig	Neutralizing activity of FMDV *in vitro* and delayed clinical symptoms and transmission *in vivo*.	(39)
	scFv	Vero	Pig	Antigen targeting of ASFV to SLA.	(40)
	Chimeric M x P Full Ab	Yeast	Pig	Growth inhibition of *H. parasuis in vitro* and partial protection *in vivo* to prevent Glasser's disease.	(41)
	scFv	*E. coli*	Cow	Blocks cell adhesion of ETEC. Reduced ETEC infection *in vivo*.	(42)
	scFv	*E. coli*	Cow	Inhibits cell adhesion of ETEC by blocking K99 factor, evaluated *in vitro*.	(43)
	scFv	Plants	Cow	Diminished ETEC binding ability in calf enterocytes and in horse blood red cells.	(44)
	scFv	*E. coli*	Chicken	Neutralizing activity against the IBV.	(45)
	scFv	*E. coli*	Chicken	Reduced viral infection of IBDV *in ovo*.	(46)
	scFv	LMH^*^ cells	Chicken	Decreased viral titers *in vivo* of IBDV.	(47)
	scFv	*E. coli*	Chicken	Neutralization of NDV, low viral titers and cytopathic effect *in vitro*.	(48)
	scFv	Plants	Chicken	Diminished oocyst count of *E. tenella* (coccidia) in feces.	(49)
	Full Ab	Plants	Chicken	Recognizes oocysts, sporocyst walls and sporozoites antigens of *E. acervulina*.	(50)
	scFv	Plants	Chicken	Diminished oocysts count of *E. tenella* in feces.	(51)
	scFv	HEK293	Sheep	Improved cellular IFN response to Rift Valley Fever virus through antigen targeting.	(52)
Therapeutics	Full Ab	HEK293	Pigs	Recognition of groups 1 and 2 hemagglutinins and neutralizing activity against influenza virus.	(53)
	Full Ab	CHO	Pigs	Reduced gross pathology in lungs after challenge with influenza A virus. No effect on viral titers.	(54)
	scFv	*E. coli*	Cow	Obstruction of tissue adhesion by *S. aureus* to prevent bovine mastitis.	(55)
	sdAb (VHH)	*E. coli*	Cow	Recognizes β-hemolysin of *S. aureus*. Shows neutralizing activity *in vitro*.	(56)
	Chimeric M x C Full Ab	CHO	Chicken	Recognition of VP2 protein of virulent strain of IBDV, 80% protection in chickens.	(57)
	sdAb	*E. coli*	Chicken	Neutralizing activity specific for the H5 hemagglutinin from AIV. High protection rates *in vivo*.	(58)
	Fab	*E. coli*	Chicken	Neutralization and inhibition of hemagglutination in infected mice.	(59)
	scFv	*E. coli*	Chicken	Reduced viral titers of avian influenza H5N1 virus.	(60)
	Chimeric M × D Full Ab	CHO	Dog	Effective to treat chronic inflammation in dogs.	(61)
	Caninized Full Ab	CHO	Dog	Treatment of atopic dermatitis, reduction of pruritus.	(62, 63)
	scFv	CHO and *E. coli*	Dog	Anti-canine CD20, potential use in the treatment and diagnosis of B cell malignancies.	(64)
	Caninized Full Ab	CHO	Dog	Recognizes EGFR. Antiproliferative effect of cancer cells *in vitro*.	(65)
	scFv	*E. coli*	Dog	Potential use for targeting to canine dendritic cells.	(66)

* *LMH, Leghorn male hepatoma cell line*.

## Recombinant antibodies in veterinary diagnosis

### Pig

Research for new diagnostic tests using rAbs have been concentrated on porcine circovirus type II (PVC2), classic swine fever virus (CSF), *Brachyspira hyodysenteriae* and *Taenia* spp. A commercial PVC2 vaccine was used to immunize a camel and produce a sdAb anti-Cap protein (11). This sdAb showed high specificity and sensitivity for the detection of PVC2 without cross reactivity with PCV1, porcine reproductive and respiratory syndrome virus (PRRSV) GP5 protein or CSF E2 protein. The sdAb was fused with alkaline phosphatase (sdAb-AP) to improve diagnosis, and the affinity and sensitivity were higher than those of the original sdAb (12). Recently, a porcinized rAb anti-E2 protein of CSFV was produced. This porcinized rAb was evaluated in diverse assays with good results and importantly, retained the ability to neutralize CSFV *in vitro*, suggesting that it has great potential as a diagnostic tool (13). Lobova et al. (14) described a scFv capable of detecting *B. hyodysenteriae*, an etiological agent of swine dysentery. After ELISA and immunofluorescent assay (IFA) evaluation, the authors concluded that this scFv can be used in new diagnostic tests (14). In the case of parasitic diseases, a sdAb showed no cross reactivity with *T. saginata, T. hydatigena, T. crassiceps*, and *Trichinella spiralis* antigens, allowing the specific diagnosis of *Taenia solium* infection (15).

### Cows

The rAbs for the most important pathogens affecting bovines, including foot and mouth disease virus (FMDV), *Mycobacterium bovis and* bovine spongiform encephalopathy (BSE), have been evaluated by new diagnostic tests. Two scFvs specific for the 3ABC antigen have been reported to differentiate between vaccinated and infected animals with FMDV. Foord et al. (16) produced a chicken scFv specific for the 3B region of the 3ABC antigen and concluded that scFv could be used in a FMDV differentiating infected from vaccinated animals (DIVA) test offering superior results compared with those of the 3ABC antigen in an ELISA, using sera from naïve and infected animals (16). Sharma et al. (17) validated a scFv in sandwich and competitive ELISAs and proposed it as an alternative to the diagnosis of FMDV (17). Other authors have produced a scFv anti-VP1 protein of FMDV in transgenic Tobacco plants (18). In the case of the bovine immunodeficiency virus (BIV), a scFv anti-capsid protein was produced and showed better sensitivity in ELISA and Western blot (WB) assays than did the mAb, which is considered the “gold standard” (19). A scFv anti-HSP65) protein was conjugated to colloidal gold and evaluated in immunochromatographic tests (ICT) as a capture/secondary antibody to improve *M. bovis* diagnosis. This combination could detect the HSP65 protein dimer by ICT but not by ELISA. Later, this scFv was fused with a chicken H chain, and the stability was improved without affecting functionality (20). In another study, a sdAb produced in camels immunized with intracellular bacteria *Brucella melitensis*, which is responsible for Mediterranean fever in animals and humans, could recognize antigens of a *B. melitensis* in ELISA (21). Finally, recombinant chicken immunoglobulin Y (IgY) (Ab3-15 and Ab4-19) anti-prion protein (PrP^sc^) was produced to diagnose BSE, showing that it can be used to diagnose BSE and other prion diseases (22).

### Others

In poultry, a scFv specific for the avian influenza virus (AIV) was produced and evaluated by ELISA; it showed higher sensitivity and specificity than previously established protocols (23). Additionally, scFv anti-phosphoprotein of the Newcastle disease virus (NDV) showed potential as a detection tool in ELISA and WB (24). A similar scenario was reported for a scFv specific to the infectious bursal disease virus (IBDV) (25, 26) and a scFv specific to avian coccidiosis (27). Taking advantage of the similarities between the human epidermal growth factor receptor 2 (HER2) and its canine homologue protein, dog epidermal growth factor receptor (DER2), two rAbs with cross reactivity were produced, a Fc-sdAb and a GFP-sdAb. Recognition of HER2 and DER2 was evaluated by flow cytometry and IFA to detect breast cancer cells from humans (SKBR3) and dogs (SH1B and P114). Therefore, these rAbs can be used to identify malignant cells and have the potential to be used as immunotherapeutics in dogs and humans (28).

## Recombinant antibodies in immunoprophylaxis

The amount of research on rAb as a new immunoprophylaxis for pigs has been enormous compared with that for bovine or other species. Different forms of rAb for porcine epidemic diarrhea virus (PEDV), PRRSV, FMDV, African swine fever virus (ASFV), and *Haemophilus parasuis* have been produced and evaluated. However, the majority of rAbs have been directed to PRRSV control.

### Pigs

Antigen targeting to antigen-presenting cells, such as dendritic cells (DCs), has become an attractive approach in veterinary medicine (67). Other authors have evaluated this strategy but not with rAb (68–70). Subranamiam et al. has probed a scFv-Fc (mouse x pig) specific to DC-SIGN, DEC205 and Langerin receptors, and fused it with PRRSV structural proteins. The results showed that DEC205 targeting is the most successful in improving humoral and cellular responses while not inducing enough protective immunity (29, 30). In a similar approach, the administration of a rAb conjugated to peptides of PRRSV glycoprotein 4 (GP4) was used to target sialoadhesin (CD169), a receptor present in macrophages and monocytes. This study showed the production of anti-GP4 and neutralizing antibodies in immunized and challenged pigs (31). In other strategies, a sdAb specific to the non-structural protein 9 (nsp9) (Nb6) was produced to block viral infection. The authors demonstrated that viral replication was inhibited in a stable Marc-145 cell line expressing Nb6, proving the potential of sdAb (Nb6) as a new form of protection against PRRSV (32). Similarly, a chimeric mouse x pig antibody anti-linear GP5 epitope had neutralizing activity similar to that of the native mAb (ISU25C1) (33).

Certainly, most rAbs reported today are used to seek control of PRRSV. However, there are also other rAbs that can be used to control other diseases. A scFv-Fc (mouse x pig) specific for the Langerin receptor expressed on DCs was fused with the spike protein of PEDV and used to immunize pigs. This strategy induced IgG and IgA responses, as well as a CD4 T cell immune response (34). When this rAb was tested in sows, an IgG response but not an IgA response was produced. Piglets born from these sows were challenged, and maternal immunity reduced fecal viral shedding, while clinical signs were unaffected (35). In other reports, a scFv anti-PEDV was tested *in vitro* and showed neutralizing activity, a reduced cytopathic effect and a reduction in viral replication titers (36). Similarly, a scFv against porcine aminopeptidase N (pAPN) could block the interaction of PEDV with pAPN, the receptor present in the intestinal epithelium, thus inhibiting virus entry to cells (37).

FMDV has also been a focus of rAb production. Harmsen et al. (71) evaluated a sdAb (VHH) as a tool to control FMD in pigs (71). Subsequently, different forms of sdAbs (VHH2s and VHH3s) were produced to increase its half-life and neutralizing activity compared with those of VHH *in vitro*. In an *in vivo* experiment, the authors compared VHH2s and VHH3s forms, showing the advantages of VHH3 forms; VHH3s delayed the development of clinical symptoms and FMDV transmission, basically for the doses, route of administration and higher neutralizing activity (38, 39). In an effort to control ASFV, a DNA vaccine encoding a scFv fused with p54 and p30 antigens was evaluated. Unfortunately, this strategy was unable to induce protection following a challenge (40).

For bacterial infections, only one rAb has been reported. This rAb (chimeric mouse × pig antibody) recognizes all serotypes of *Haemophilus parasuis*, inhibits the growth of the bacteria *in vitro*, and partially protects piglets against infection *in vivo* (41).

### Cows

In contrast to those concerning pigs, there are fewer reports of rAbs being used to improve bovine diseases. Several attempts have been made to block *Escherichia coli* enterotoxigenic (ETEC) cell-adhesion capacity by targeting K99 fimbriae, a colonization factor. An *in vivo* evaluation of a scFv anti-F5 fimbriae (K99) resulted in less accumulation of fluid in the intestinal loops, indicative of a reduced ETEC infection in neonatal calves (42). Moreover, similar scFvs directed at K99 factor have been evaluated *in vitro* using horse red blood cells and calf enterocytes (43, 44) where hemagglutination and binding activity has been reduced.

### Others

A scFv anti-infectious bronchitis virus (IBV) showed neutralizing activity (45); similarly, a scFv-Fc and scFv anti-infectious bursal disease virus (IBDV) reduced viral titers *in vivo* and *in ovo* (46, 47). NDV is a highly contagious viral infection that affects poultry and domestic birds. A scFv that recognizes NDV was produced and evaluated *in vitro*; the Ab showed neutralizing activity that resulted in reduced viral titers, as well as a lower cytopathic effect, in BHK21-infected cells (48). A scFv anti-*Eimeria tenella* and -*E. acervulina* produced in pea plants and *Nicotiana benthamiana*, respectively (49, 50), reduced the number of oocysts in the feces of chickens fed with transformed plants, especially when feeding with pea plants (51). Additionally, IgA rAb anti-*E. acervulina* antigens have been produced in *N. benthamiana* plants as potential immunotherapy agents for young broilers in which vaccination is not always successful (50).

In sheep, a DNA vaccine encoding a scFv fused with Rift Valley Fever Virus Gn peptide was directed at DEC205 and CD11c receptors. Compared with targeting to CD11c and untargeted treatment, targeting to DEC205 promoted IFNγ production but showed an inefficient humoral response (52).

## Recombinant antibodies for therapeutic purposes

The use of rAb for therapy has been mainly focused on dogs where personalized treatments are less challenging than those for farm animals. Examples include therapies for cancer and inflammatory processes.

### Pigs

An anti-influenza virus antibody was obtained from a human donor after infection with H1N1 swine-origin influenza virus (SOIV). From this donor, a rAb named F16 was expressed and evaluated in several animal models. In mice and ferrets, F16 showed a therapeutic effect after a lethal challenge with H1N1 and H5N1 viruses, respectively (53). Nonetheless, when evaluated in pigs, F16 did not alter viral titers, although it reduced gross lung pathology when challenged (54).

### Cows

Bovine mastitis is caused by a variety of pathogens including *Staphylococcus aureus*. Tissue adhesion involves multiple proteins, including fibronectin-binding factor A and clumping factor A. These two proteins were targeted using a scFv to obstruct the adhesion mechanisms; therefore, they have potential to be used to prevent and treat bovine mastitis (55). Similarly, a sdAb that could recognize and neutralize β-hemolysin from *S. aureus* was generated and evaluated *in vitro* to confirm its neutralizing activity (56).

### Others

Therapy in poultry is, in most cases, not economically practical. However, the development of rAbs can simplify this practice. In this manner, a full-IgY rAb that can neutralize IBDV provides an 80% protection rate, in contrast to yolk antibody that offers only 40% protection in chickens challenged with IBDV (57). Similarly, a recombinant adenovirus (Ad5) containing the encoding sequence of a neutralizing sdAb specific for the HA1 domain of H5 virus was created. *In vivo* administration showed a 90–100% survival rate in lethally challenged mice (58). Additionally, a Fab that recognizes distinct HA0 hemagglutinin epitopes showed neutralizing activity and has great therapeutic potential (59). In this manner, a scFv anti-HA fused with truncated protamine (scFv-tP) designed to deliver siRNA was produced (scFv-tP) and evaluated *in vitro*, showing a reduction in viral titers (60).

In some cases, human medicine can be adapted for use in veterinary medicine. An example is a chimeric dog x mouse rAb derived from a mouse anti-human nerve growth factor (NGF) mAb (72) used to treat chronic inflammation. *In vivo*, a single dose of this rAb showed effectiveness in reducing chronic pain similar to that of 7 daily doses of meloxicam in dogs, proving it to be a good alternative for prolonged therapeutic treatments (61). A full caninized rAb anti-canine IL-31, lokivetmab, has been produced and evaluated in clinical trials with outstanding results concerning the control of atopic dermatitis (AD) in dogs (62). Compared to a daily dose of 5 mg/kg of ciclosporin, a drug typically used to treat AD, a single dose of 1 mg/kg ameliorated the symptoms of AD for a month (63). In the field of cancer, a scFv anti-canine CD20, a cell surface molecule expressed in normal and tumoral cells, was produced (64). Similarly, a caninized full Ab against epidermal growth factor receptor (EGFR) induces significant inhibition in proliferation *in vitro* and viability reduction in canine tumor cells that overexpress EGFR (65). Another anti-tumoral strategy is DC-based vaccination; a scFv capable of recognizing canine DCs has been produced for future use in vaccination and therapy (66).

## Conclusion

The continuous search for alternative ways to control pathogens influenced the application of rAbs in veterinary medicine, which appears to be influenced by the type of animal production. Most reports describing the use of rAbs for diagnosis are concentrated in cows. Their use for treatment is concentrated in dogs, and use for immunoprophylaxis is concentrated in pigs. Unsurprisingly, in the case of pigs, most reports are concentrated on a solution for PRRSV; alternatively, therapeutic rAb is used to identify treatments for dog diseases (Table 1). There is a wide diversity of rAb forms (Figure 1), and not all have been described in veterinary medicine. However, scFv, chimeric and sdAb are the most common forms of rAbs reported to date. In the upcoming years, the use of rAbs for the control of animal diseases will be a reality and will become a significant part of the economic world of pharmaceuticals. In summary, for the first time, the present mini-review describes the progress of rAb use in the field of veterinary medicine. This technology has not been fully exploited for diseases of economically impactful animals. The development of rAbs has been proven to be a promising tool in the improvement of animal health.

## Author contributions

All authors listed have made a substantial, direct and intellectual contribution to the work, and approved it for publication.

### Conflict of interest statement

The authors declare that the research was conducted in the absence of any commercial or financial relationships that could be construed as a potential conflict of interest.
